# Identification of Hub Genes in Colorectal Adenocarcinoma by Integrated Bioinformatics

**DOI:** 10.3389/fcell.2022.897568

**Published:** 2022-05-27

**Authors:** Yang Liu, Lanlan Chen, Xiangbo Meng, Shujun Ye, Lianjun Ma

**Affiliations:** ^1^ Endoscopy Center, China-Japan Union Hospital of Jilin University, Changchun, China; ^2^ Department of Hepatobiliary and Pancreatic Surgery, The First Hospital of Jilin University, Changchun, China

**Keywords:** colorectal adenocarcinoma, differential gene expression analysis, weighted gene co-expression network analysis, tumor biomarkers, predictive model

## Abstract

An improved understanding of the molecular mechanism of colorectal adenocarcinoma is necessary to predict the prognosis and develop new target gene therapy strategies. This study aims to identify hub genes associated with colorectal adenocarcinoma and further analyze their prognostic significance. In this study, The Cancer Genome Atlas (TCGA) COAD-READ database and the gene expression profiles of GSE25070 from the Gene Expression Omnibus were collected to explore the differentially expressed genes between colorectal adenocarcinoma and normal tissues. The weighted gene co-expression network analysis (WGCNA) and differential expression analysis identified 82 differentially co-expressed genes in the collected datasets. Enrichment analysis was applied to explore the regulated signaling pathway in colorectal adenocarcinoma. In addition, 10 hub genes were identified in the protein–protein interaction (PPI) network by using the cytoHubba plug-in of Cytoscape, where five genes were further proven to be significantly related to the survival rate. Compared with normal tissues, the expressions of the five genes were both downregulated in the GSE110224 dataset. Subsequently, the expression of the five hub genes was confirmed by the Human Protein Atlas database. Finally, we used Cox regression analysis to identify genes associated with prognosis, and a 3-gene signature (CLCA1–CLCA4–GUCA2A) was constructed to predict the prognosis of patients with colorectal cancer. In conclusion, our study revealed that the five hub genes and CLCA1–CLCA4–GUCA2A signature are highly correlated with the development of colorectal adenocarcinoma and can serve as promising prognosis factors to predict the overall survival rate of patients.

## Introduction

With the increasing incidence rate of colorectal cancer (CRC) worldwide, it is considered as one of the leading causes of cancer deaths (Siegel et al., 2020). It is expected to cause about 53,200 deaths by 2020 (Colorectal Cancer Statistics | How Common Is Colorectal Cancer? 2021). Recently, combined therapies including surgery, chemotherapy, targeted therapy, and radiotherapy have prolonged the overall survival (OS) of patients with colorectal cancer (van der Geest et al., 2015). However, distant metastasis and drug resistance are still the main reasons for the poor prognosis effect of colorectal cancer patients. At present, the exact carcinogenic molecular mechanism of colorectal cancer is not precise, and no effective prognostic biomarkers have been thoroughly investigated. Therefore, it is necessary to explore the molecular mechanism of the proliferation and progression of colorectal cancer to find promising prognostic biomarkers and formulate effective clinical treatment strategies.

With the rapid development of sequencing technology, bioinformatics is increasingly widely used in gene expression profiling to study the molecular mechanism of diseases and find disease-specific biomarkers (Can, 2014). Weighted gene co-expression network analysis (WGCNA) is an effective tool to construct the related networks and identify hub genes, which is widely used to find tumor biomarkers (Langfelder and Horvath, 2008). Highly related genes may be functionally related and can be clustered into a module by WGCNA. The correlation between modules with clinical characteristics can be quantified and helps to identify modules of interest. In addition, differential gene expression analysis can also provide an essential method for studying the molecular mechanism of genome regulation and revealing the quantitative changes in expression levels between the experimental and control groups, which might help us find new colorectal cancer biomarkers. Therefore, two methods are used, combining the results of WGCNA and differential gene expression analysis to enhance the recognition ability of highly related genes, which is helpful to be used as candidate biomarkers (San Segundo-Val and Sanz-Lozano, 2016).

In this study, WGCNA and differential gene expression analysis was performed to analyze the mRNA expression data of COAD-READ in TCGA and GEO databases, and the differentially co-expressed genes were obtained. We further explored the biological function of these differentially co-expressed genes of COAD-READ using functional enrichment and protein interaction (PPI) analysis combined with survival analysis. This study provides a possible basis to understand the regulating mechanism of COAD-READ by analyzing differential co-expression genes and provides a novel 3-gene signature for clinical diagnosis or treatment.

## Materials and Methods

### Datasets From TCGA and GEO Databases

COAD-READ gene expression profiles were downloaded from TCGA (https://portal.gdc.cancer.gov/) and GEO (https://www.ncbi.nlm.nih.gov/gds) databases. In TCGA database, all COAD-READ data and clinical information can be downloaded through R package TCGA biolinks (Colaprico et al., 2016). A total of 444 COAD-READ samples were collected, including 404 colorectal adenocarcinoma and 40 normal tissues, consisting of the raw read count data of 19,600 genes. According to the suggestion of the edgeR package manual, lowly expressed genes are usually considered noise in differential expression analysis (Robinson et al., 2010). Therefore, only genes with ≥1 CPM (count per million) were retained for downstream analysis. Then, the expression level of 14,091 genes was calculated using the rpkm function in the edgeR package. In addition, the standardized expression profile of GSE25070, another gene expression profile of COAD-READ in the GEO, was obtained by R package GEO query (Davis and Meltzer, 2007). GSE25070 was composed of the expression profiles of 26 tumor samples from patients with COAD-READ and 26 pairs of normal tissues. The GPL6883 platform Illumina HumanRef-8 v3.0 expression BeadChip was used to study GSE25070. The probes were converted into gene symbols according to the annotation file provided by the manufacturer, and the duplicate probes of the same gene were removed by determining the expression median of all corresponding probes. In the end, 18,599 genes were chosen to be further analyzed.

### Using WGCNA to Identify Key Co-Expression Modules

The co-expression network facilitates gene screening technology, which can be used to identify potential biomarkers and treatment targets. Our study built the gene expression profiles of TCGA COAD-READ and GSE25070 into a weighted gene co-expression network by WGCNA package (Langfelder and Horvath, 2008). WGCNA was used to analyze the highly correlated gene modules among samples, and the gene modules related to the external traits of samples were discussed. The pickSoftThreshold function is used to help with selection β to ensure a scale-free network (Zhang and Horvath, 2005). Next, the topological overlap matrix (TOM) and the corresponding dissimilarity matrix (1-TOM) are calculated by using the obtained adjacency matrix. In order to further identify the functional modules in the co-expression network, the module feature association and clinical trait information between the modules were calculated according to the previous studies. We calculated the correlation between modules and clinical data to determine the crucial clinical module. Therefore, the module with a high correlation is considered the candidate associated with clinical traits and chosen to analyze later.

### Differential Gene Expression Analysis and Interaction With the Important Modules

R package limma was used to identify differentially expressed genes (DEGs) in TCGA COAD-READ dataset and the microarray data of GSE25070. Limma is an R/Bioconductor software package, which provides an integrated solution for analyzing the data of gene expression (Ritchie et al., 2015). Astringent filter of |logFC| ≥ 1.0 and *p* < 0.05 was applied to identify reliable DEGs. Then, R package “ggplot2” (http://ggplot2.org) was further used to draw the volcanic map of all DEGs between COAD-READ and the control group, and R package “pheatmap” (https://CRAN.R-project.org/package=pheatmap) was utilized to draw the clustering heatmap of DEGs. Then, the overlapping genes between co-expression genes and DEGs extracted from the co-expression network were aggregated to explore candidate marker genes and plotted using the R package Venn diagram (Chen and Boutros, 2011).

### Functional Annotation Analysis of Significant Genes

R package clusterProfiler was used to perform KEGG and Gene Ontology (GO) enrichment analysis to explore the possible biological function of the selected genes, and *p* < 0.05 was considered statistically significant (Yu et al., 2012). All the molecular functions (MF), cellular components (CC), and biological processes (BP) were analyzed (Gene Ontology Consortium, 2006).

### Construction of a PPI Network and Screening of Hub Genes

Our study used the online tool STRING (search tool for interacting genes, version 11.0), which is designed to predict protein–protein interaction (PPI), to construct a PPI network of candidate genes (Szklarczyk et al., 2019). The pairing with the PPI score ≥0.4 was reserved, and the PPI network was constructed using Cytoscape software 3.40 (www.Cytoscape.org). Using a plug-in cytoHubba of Cytoscape, the top 10 hub genes are predicted based on the maximum clique centrality (MCC) algorithm in the co-expression network (Chin et al., 2014).

### Analysis of the Potential Prognostic Values of the Screened hub Genes

All hub genes were divided into two groups according to their expression levels. Based on Kaplan–Meier analysis, the OS curve was drawn by the survival package. (http://cran.rproject.org/package=survival). In addition, the online tool GEPIA2 was used to determine the association between disease-free survival (DFS) and hub gene expression in COAD-READ patients (Tang et al., 2019). *p* < 0.05 was considered statistically significant. Subsequently, we analyzed and compared the expression of the survival-related genes in GSE110224 and drew a box plot graph. Moreover, we performed a correlation analysis between the survival-related genes and clinical factors.

### Analysis of Survival-Related Hub Gene Protein Expression in the HPA Database

The Human Protein Atlas (HPA) database aims to map all human proteins in cells, tissues, and organs using various omics techniques (HPA; http://www.proteinatlas.org) (Zhang and Horvath, 2005; Uhlén et al., 2015; Thul et al., 2017). The HPA online tool has helped thousands of biomedical and disease researchers. Using the HPA database, protein levels of survival-related genes were detected by immunohistochemistry (IHC), and IHC images were obtained from the HPA database.

### Gene Signature Identification and Risk Score Calculation

In order to identify multiple gene features with good prognostic performance, we randomly divided the samples into the training set and the test set. Univariate Cox regression analysis was used to screen differentially expressed genes related to patient OS in the training set for survival-related genes screened in the previous step. Genes with *p* value <  0.05 are used as candidate variables, and the genes with the lowest Akaike information criterion (AIC) value are retained in the final signature. The risk coefficients of these genes were calculated using a multivariable Cox proportional risk model based on “survival” (https://CRAN.R-project.org/package = survival) and “survminer” (https://github.com/kassambara/survminer) packages. The patients were divided into high-risk and low-risk groups according to the median risk score. The Kaplan–Meier method was used to analyze the OS of the two groups, and we verified the model’s predicted value by drawing the receiver operating characteristic curves (ROCs) with 5 years of the training set, the test set, and the entire set. The ROC curve analysis was obtained by using the nearest neighbor estimation (NNE) method in “survivalROC” package (https://CRAN.R-project.org/package=survivalROC).

## Results

### Weighted Gene Co-Expression Network Analysis of the Collected CRC Datasets

**FIGURE 1:**
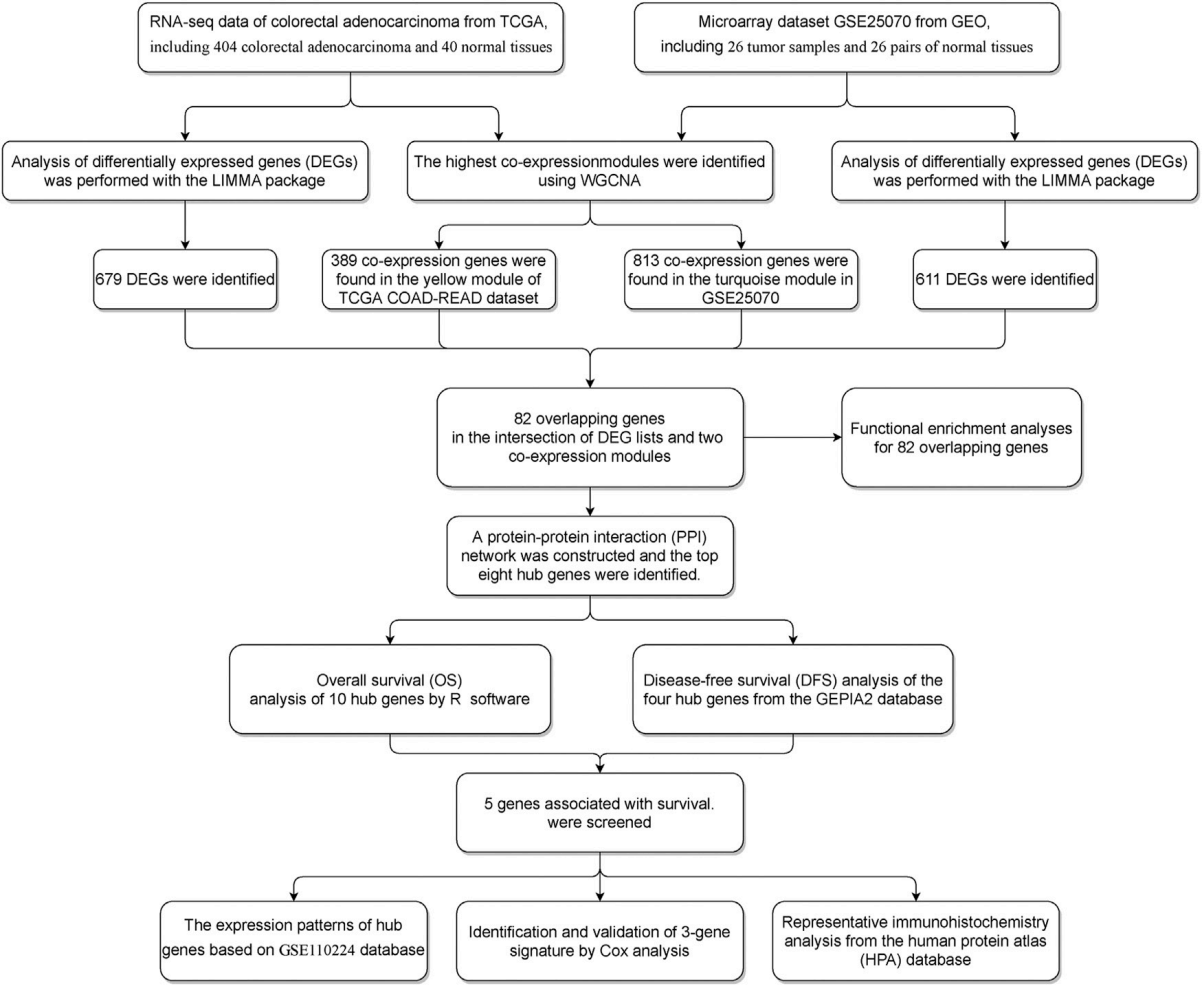
Workflow of the study design.

**FIGURE 2:**
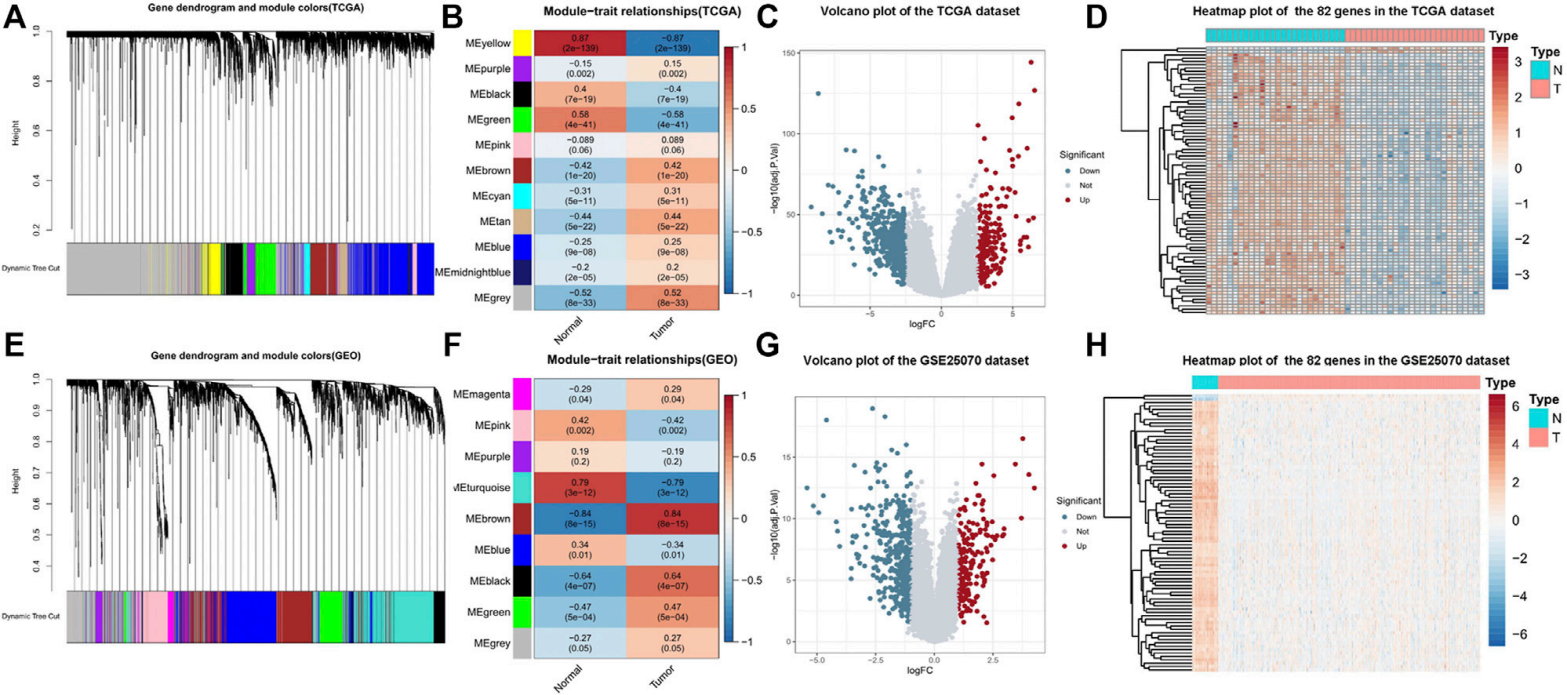
**(A)** In TCGA COAD-READ dataset, the dendrograms of all differentially expressed genes were clustered based on the measurement of dissimilarity (1-TOM). Each module was assigned a color. **(B)** In TCGA COAD-READ dataset, module-trait relationships. Each row corresponds to a module feature gene (ME), and the column corresponds to a feature. Each cell contains the corresponding correlation and the *p* value. **(C)** Volcano plot of DEGs from TCGA. **(D)** Heatmap of 82 genes in TCGA dataset. **(E)** In the GSE25070 dataset, the dendrograms of all differentially expressed genes were clustered based on the measurement of dissimilarity (1-TOM). Each module was assigned a color. **(F)** In the GSE25070 dataset, module-trait relationships. Each row corresponds to a module feature gene (ME), and the column corresponds to a feature. Each cell contains the corresponding correlation and the *p* value **(G)** Volcano plot of DEGs from the GSE25070 dataset. **(H)** Heatmap of the 82 genes in the GSE25070 dataset.

To explore the gene expression landscape in CRC patients, TCGA COAD-READ dataset and the GSE25070 dataset were downloaded and re-analyzed (Figure 1, Methods). To find the functional clusters of COAD-READ patients, WGCNA package in R was used to construct the gene co-expression network from TCGA COAD-READ (Figure 2A) and the GSE25070 dataset (Figure 2E). The research shows that the co-expression network conforms to the scale-free network: the log(k) value of node connectivity K is negatively correlated with the log(P (k)) value of node probability, and the correlation coefficient is greater than 0.85 for TCGA COAD-READ dataset and 0.8 for the GSE25070 dataset. In order to ensure that the network is scale-free, we chose β = 13 (sft$powerEstimate = 13) for TCGA COAD-READ dataset and β = 10 for the GSE25070 dataset. According to the adjacency matrix, the topological overlap matrix is generated based on the TOM similarity algorithm. Then the genes are hierarchically clustered based on this algorithm, and the minimum number of genes was set in a single gene network module to 50. After the gene modules are determined by the dynamic cutting method, the eigengenes of each module are calculated, and each module is analyzed by cluster analysis. Then, we cluster the modules and merge the highly correlated modules into a new module using the mergeCloseModules function with cutHeight set to 0.25. After merging similar modules, we can identify nine modules in TCGA COAD-READ and eight modules in GSE25070 (exclude gray modules that are not assigned to any cluster), and each module uses different colors to distinguish among them. In addition, we explored the module-trait relationship and plotted the heatmap. In TCGA dataset, the yellow (389 genes, *p* value = 2E−139), black (418 genes, *p*-value = 7E−19), and green (418 genes, *p* value = 4e−41) modules are relevant modules with normal traits, while the brown (611 genes, *p* value = 1E−20), tan (389 genes, *p* value = 2E−139), and gray modules (389 genes, *p* value = 2E−139) are relevant modules with colorectal adenocarcinoma traits. Furthermore, in the GEO dataset, the turquoise (813 genes, *p* value = 3E−12), pink (353 genes, *p* value = 0.002), and blue (689 genes, *p* value = 0.01) modules are relevant modules with normal traits, while the brown (695 genes, *p* value = 8E−15), black (212 genes, *p* value = 4E−7), and green modules (250 genes, *p* value = 5E−4) are relevant modules with colorectal adenocarcinoma traits. In particular, the results (Figures 2B,F) showed that the yellow module in TCGA COAD-READ and the turquoise module in GSE25070 had the highest negative correlation with colorectal adenocarcinoma (yellow module: *r* = - 0.87, *p* = 2 × 10^–139^ and turquoise module: *r* = - 0.79, *p* = 3 × 10^–12^).

### Identification of Genes Between the DEG Lists and Co-Expression Modules

**FIGURE 3:**
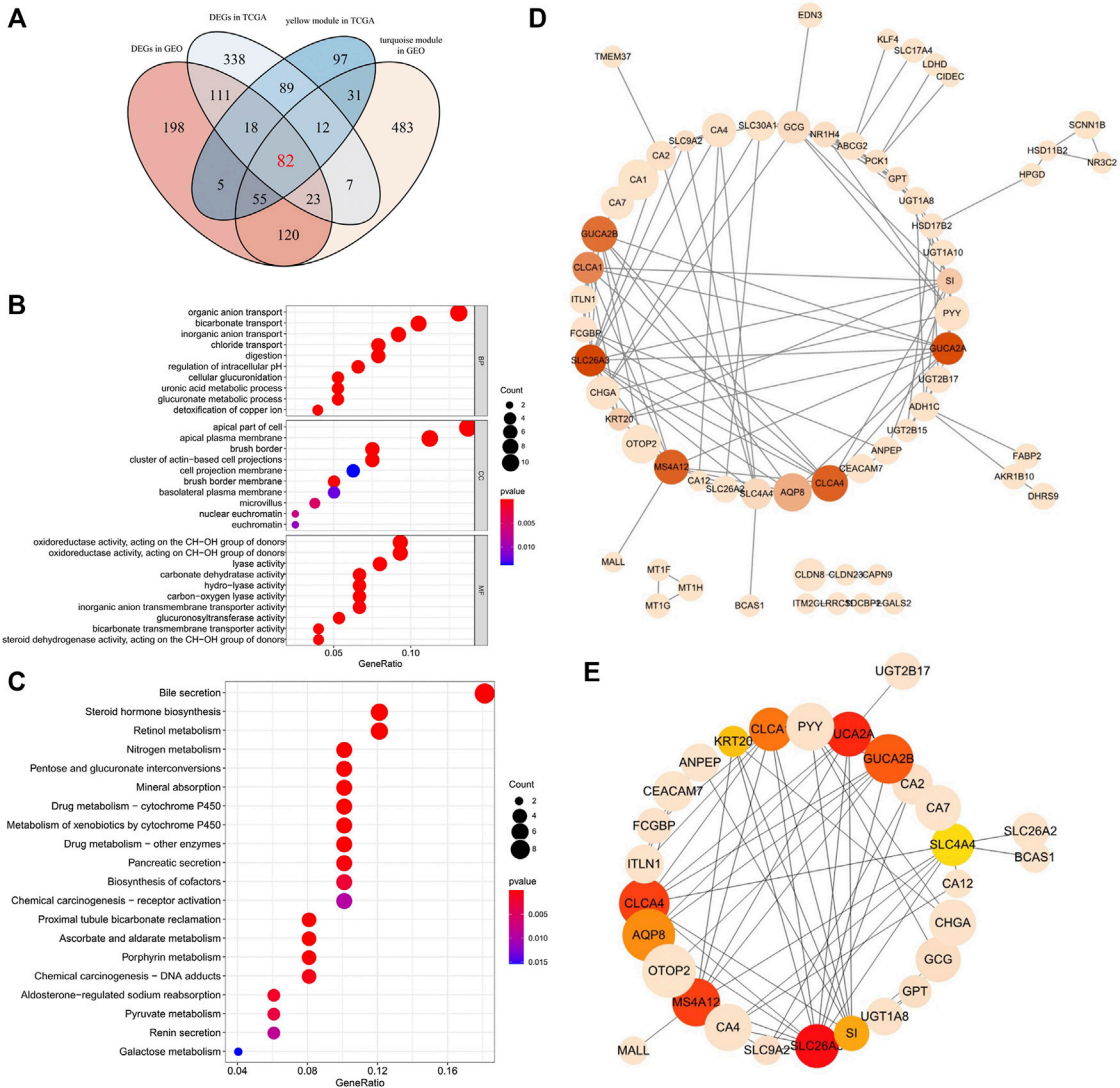
**(A)**Venn diagram of DEGs and the gene list from the co-expression module. A total of 82 overlapping genes in the intersection of DEGs and two gene lists from the co-expression module. **(B)** Gene Ontology (GO) enrichment analysis for the 82 overlapping genes. The color of each bubble represents the *p* value, and the bubble size represents the number of genes. **(C)** KEGG pathways enrichment analysis for the 82 overlapping genes. The color of each bubble represents the *p* value, and the bubble size represents the number of genes. **(D)** PPI network of the 82 overlapping target genes. There were 82 nodes, where nodes represented genes, and edges were the interactions between two genes. Red is the higher score calculated by the MCC method, followed by yellow, and the size of nodes corresponds to absolute logFC values. **(E)** A total of 10 hub genes were identified from 82 genes *via* MCC algorithm analysis. Network nodes represent proteins; edges represent protein–protein associations. Red is the higher score calculated by the MCC method, followed by yellow, and the size of nodes corresponds to absolute logFC values.

According to the cutoff criteria of |logFC| ≥ 2.5 and FDR <0.01 for TCGA dataset and |logFC| ≥ 1.0 and FDR <0.05 for the GSE25070 dataset, 679 differentially expressed genes in TCGA dataset (Figure 2C) and 611 in the GSE25070 dataset (Figure 2G) were identified between COAD-READ and normal tissues. As showcased in Figure 3A, 389 and 813 co-expression genes were found in the yellow module of TCGA COAD-READ dataset and the turquoise module in GSE25070, respectively, 82 overlapping genes (Supplementary Table S1) were extracted, of which 80 were upregulated, and two were downregulated. Then, the expression pattern of these differentially expressed genes in the COAD-READ and GSE25070 datasets are plotted in Figures 2D,H, respectively.

### Functional Enrichment Analyses for the 82 Extracted Genes

To further explore the biological function of the 82 differentially expressed genes, the clusterProfiler package (Yu et al., 2012) in R was used to perform GO and KEGG enrichment analyses (Ashburner et al., 2000; Gene Ontology Resource, 2021). The results of the GO enrichment analysis are provided in Figure 3B. In the biological processes (BP) category, many terms are related to the metabolism and transport of substances, such as bicarbonate transport and cellular glucuronidation. Moreover, related studies suggest that bicarbonate transporter may change the proliferation rate of cancer cells by regulating the pH value of cancer cell cytoplasm and extracellular space (Gorbatenko et al., 2014). Furthermore, glucuronidation is an essential metabolic pathway of many small endogenous and exogenous lipophilic compounds, including bilirubin, steroid hormones, bile acids, and carcinogens (Hu et al., 2014). Cellular components (CC) showed that these genes were mainly distributed in the apical part of the cell and brush border. The primary enrichment of molecular function (MF) is carbonate dehydratase activity and oxidoreductase activity. In addition, 14 distinct KEGG signaling pathways related to colorectal adenocarcinoma were identified (*p* < 0.001, Figure 3C), such as the bile secretion signaling pathway, nitrogen metabolism signaling pathway, steroid hormone biosynthesis signaling pathway, and pancreatic secretion signaling pathway. The bile secretion signaling pathway was the most significantly enriched functional category and had the highest number of enriched genes. Studies have indicated that bile acid-microbiota crosstalk exhibit a vital role in developing colorectal cancer (Louis et al., 2014; Jia et al., 2018). Interestingly, pancreatic secretion, which is also one of the secretions of the digestive glands, appears to play an essential role in the development of colorectal cancer, and some studies suggest hyperinsulinemia and type 2 diabetes mellitus (T2D) are the conditions with an increased risk of CRC (Suh and Yuspa, 2005; Winpenny et al., 2009). Most of the previously mentioned pathways may play a vital role in tumorigenesis.

### Construction of a PPI Network and Identification of Hub Genes

The PPI network of overlapping genes was established based on the STRING database, with 82 nodes and 553 edges (Figure 3D). The hub gene selected from the PPI network using the cytoHubba plug-in and the MCC algorithm is shown in Figure 3E. The top 10 hub genes were screened from the PPI network according to the MCC algorithm, including solute carrier family 26 member 3 (SLC26A3), guanylate cyclase activator 2A (GUCA2A), chloride channel accessory 4 (CLCA4), membrane spanning 4-domains A12 (MS4A12), guanylate cyclase activator 2B (GUCA2B), chloride channel accessory 1 (CLCA1), AQP8 (Aquaporin 8), sucrase-isomaltase (SI), keratin 20 (KRT20), and solute carrier family 4 member 4 (SLC4A4).

### Analysis of the Prognostic Values, Expression Patterns, and Protein Expression of Hub Genes

**FIGURE 4:**
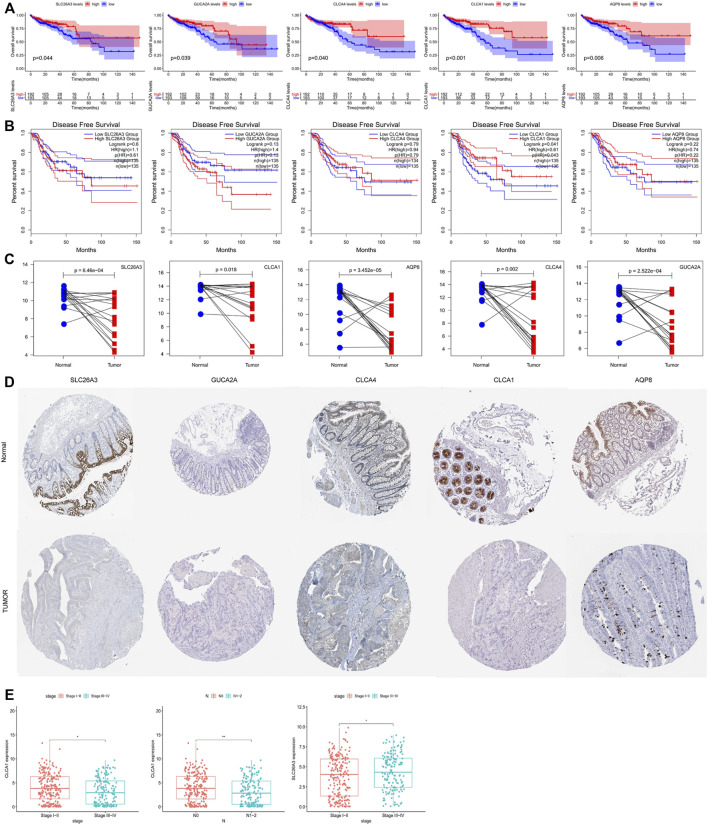
**(A)** Overall survival (OS) analysis of 10 hub genes in colorectal adenocarcinoma patients by R survival package. Patients were divided into high-level group (red) and low-level group (blue) according to the median. *p* values <0.05 were considered to be statistically significant. **(B)** Disease-free survival (DFS) analysis of the five hub genes (SLC26A3, GUCA2A, CLCA4, CLCA1, and AQP8) in colorectal adenocarcinoma patients from the GEPIA2 database. Patients were divided into high-level group (red) and low-level group (blue) according to the median. *p* values <0.05 were considered to be statistically significant. **(C)** Validation of the expression levels of 10 hub genes in colorectal adenocarcinoma patients and normal tissues in the GSE110224 dataset. **(D)** Expression profiles of the four genes in the colorectal adenocarcinoma patients and normal tissue. Images were taken from the Human Protein Atlas (http://www.proteinatlas.org). **(E)** Correlation analysis between the survival-related genes and clinical factors. *p* values ≤0.05 were considered to be statistically significant.

To study the possible clinical relevance of the identified hub gene, survival package and the GEPIA2 database were used to analyze the overall survival (OS) (Figure 4A) and disease-free survival (DFS) (Figure 4B). Among the ten hub genes, five genes were significantly associated with poor OS of the colorectal adenocarcinoma patients (*p* < 0.05); these are SLC26A3, GUCA2A, CLCA4, CLCA1, and AQP8. In addition, DFS analysis showed that CLCA1 was significantly correlated with DFS. Moreover, the previously mentioned five survival-related genes were further verified in the GSE110224 dataset. As shown in Figure 4C, all of the five hub genes were downregulated in colorectal adenocarcinoma tissues compared with adjacent normal tissues. As shown in Figure 4E, there was a significant difference in CLCA1 expression between the TNM stage and the N stage. CLCA1 expression in patients with TNM III–IV stages and N 1–2 stages was significantly decreased compared with patients with TNM I–II stages and N 3–4 stages, and SLC26A3 expression in patients with TNM I–II stages were significantly decreased compared with patients with TNM I–II stages. According to the HPA database (Figure 4D), the protein levels of SLC26A3, GUCA2A, CLCA4, CLCA1, and AQP8 in tumor tissues were all significantly lower than that in normal tissues. The aforementioned observations confirmed that the downregulation of SLC26A3, GUCA2A, CLCA4, CLCA1, and AQP8 was associated with poor prognosis and reduced overall survival in colorectal cancer adenocarcinoma patients. Moreover, top-ranking 25% of genes with the highest KME within WGCNA co-expression modules were considered significant genes, and the complete lists of gene sets and kME values are included in Supplementary Table S2 and Supplementary Table S3. We found AQP8, CLCA4, and GUCA2A were significant genes of the yellow module in TCGA dataset, and all five genes were significant genes of the turquoise module in the GSE25070 dataset.

The details of the five hub genes are as follows: chloride channel attachment 1 (CLCA1) and chloride channel attachment 4 (CLCA4) are CLCA proteins. Studies have shown that CLCA protein members affect various biological processes such as cell differentiation, adhesion, apoptosis, and airway inflammation (Elble and Pauli, 2001; Patel et al., 2009; Piirsoo et al., 2009). Both CLCA1 and CLCA4 are expressed in the intestine (Gandhi et al., 1998; Bustin et al., 2001; Jia et al., 2018; Liu et al., 2018), may act as tumor suppressors, and are negatively correlated with tumor formation (Bustin et al., 2001). Recent studies have shown that the expression levels of CLCA proteins, including CLCA1 and CLCA4, are abnormal in many cancer types so it may be a potential cancer predictor for patients (Yang et al., 2013; Yu et al., 2013; Hou et al., 2017; Liu et al., 2018). In addition, according to the literature, the mRNA levels of CLCA1 in colorectal cancer (Yang et al., 2013), ovarian cancer (Musrap et al., 2015), and pancreatic ductal adenocarcinoma (Hu et al., 2018) were different from those in normal tissues, and the loss of CLCA4 expression was observed in colorectal cancer (Chen et al., 2019), hepatocellular carcinoma (Liu et al., 2018), breast cancer (Yu et al., 2013), and bladder cancer (Hou et al., 2017). Our data showed that CLCA1 and CLCA4 were significantly downregulated in colorectal cancer compared with normal tissues. Previous studies have shown that the increase of CLCA1 and CLCA4 levels in tumor tissue is related to the excellent prognosis of colorectal cancer patients, consistent with our survival analysis (Chen et al., 2018; Pan et al., 2019a; Pan et al., 2019b; Chen et al., 2019; Wei et al., 2020a; Wei et al., 2020b).

GUCA2A is an endogenous ligand for the guanylate cyclase 2C (GUCY2C) receptor and peptide hormone and is expressed in gut epithelial cells (Blomain et al., 2013; Lima and Fonteles, 2014; Ikpa et al., 2016). Locally, it acts as autocrine and paracrine hormones to regulate GUCY2C signal transduction and humoral electrolyte homeostasis (Steinbrecher et al., 2002; Brierley, 2012). GUCY2C has a protective effect on colorectal tumors (Li et al., 2007). The downregulation of GUCA2A leads to the loss of the GUCY2C signal cascade and promotes tumorigenesis (Li et al., 2007). Previous studies have shown that the effect of the expression of GUCY2C is significant to the occurrence and development of tumors (Pattison et al., 2016; Bashir et al., 2019). This corresponds well with our findings where we showed that the down expression of GUCA2A may also contribute to colorectal adenocarcinoma carcinogenesis.

SLC26A3 is a member of the Slc26 anion transporter and channel family (Mount and Romero, 2004). SLC26A3 plays a vital role in colonic Cl−absorption (Schweinfest et al., 2006; Zhang et al., 2007). Immunohistochemical results showed that SLC26A3 was located in the apical membrane of enterocytes of the surface and crypt in the colon (Worrell et al., 2005; Schweinfest et al., 2006; Soleimani, 2006) and the apical membrane of pancreatic duct (Ishiguro et al., 2007). Consistent with the previous studies (Schweinfest et al., 1993). We found that its expression is downregulated in colorectal adenocarcinoma.

AQP8, an aquaporins (AQPs) member, is a water channel protein. Aquaporins are a family of small integral membrane proteins related to the major intrinsic protein (MIP or AQP0).

Studies show that the overexpression of AQP8 restrained CRC cell proliferation, migration, and invasion capacities *in vitro* (Wu et al., 2018). Inconsistency, AQP8 was found to be downregulated in our study.

### Identification and Validation of the 3-Gene Signature

**FIGURE 5:**
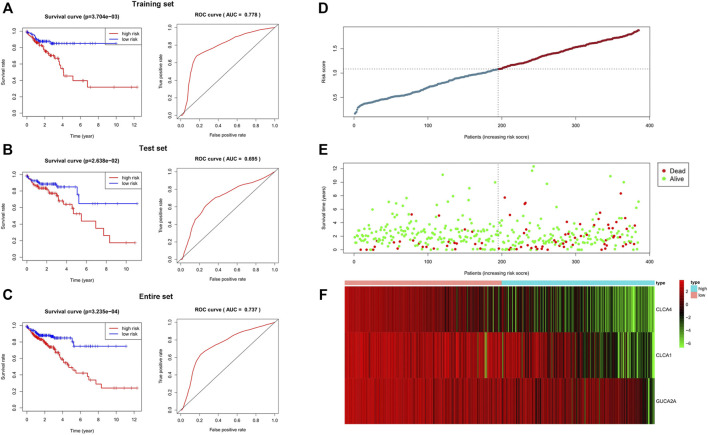
Validation of the 3-gene signature. In all three sets, there were significant differences in OS between the high-risk group and the low-risk group. The area under the ROC was 0.778 in the training set, 0.695 in the test set, and 0.737 in the entire set. The results showed that the 3-gene signature performed well in predicting the prognosis of colorectal adenocarcinoma. **(A)** Training set. **(B)** Test set. **(C)** Entire set. **(D–F)** Distributions of the 3-gene signature, survival status, and expression profiles of the three genes of entire sets.

We randomly divided 385 samples and the corresponding clinical data into a training set (n = 193) and a test set (n = 192). We found that all five hub genes were significantly associated with prognosis by univariate survival analysis in the training set. Subsequently, multivariate Cox regression analysis was used to establish a 3-gene signature. The three genes contained in this signature are CLCA1, CLCA4, and GUCA2A (Supplementary Table S4). The risk coefficients suggested that all four genes are risk factors for colorectal adenocarcinoma (coef >0). The risk score for each patient was calculated according to the following formula: risk score = (-0.053) × expression value of CLCA1 + (-0.043) × expression value of GUCA2A+ (-0.189) × expression value of CLCA4. The higher the score, the worse the patients’ prognosis were. Patients were divided into the high-risk and low-risk groups based on the median risk score. Moreover, the predicted results showed a significant difference in OS between high- and low-risk groups for all three sets (*p* < 0.05). The AUC of the ROC curve was 0.778 in the training set, 0.695 in the test set, and 0.737 in the entire set. Finally, the distribution of the risk score in each patient, survival status, and the expression of three genes are shown in Figure 5D–F.

### Comparison With Other Published Gene Signatures

We compared our 3-gene signature with other published gene signatures (Zuo et al., 2019; Gong et al., 2020; Li et al., 2020; Hong et al., 2021; Qian et al., 2021; Shao et al., 2021). In Supplementary Table S5, the AUC for 5 years OS in our 3-gene signature was 0.778 in the training set, 0.695 in the test set, and 0.737 in the entire set. The C-index of the 18-gene of Qian, 8-gene of Gong, 10-gene of Shao, 16-gene of Meng, 5-gene of Hong, and 6-gene of Zuo are 0.734, 0.713, 0.780, 0.869, 0.675, and 0.683, respectively. Compared with other prognostic models, our model has a substantial predictive value. Furthermore, our model is composed of only three genes, so it is concise and easy to use. Moreover, the three genes were obtained by the multistep screening process, including WGCNA and PPI analysis, so the 3-gene signature was not only associated with the prognosis of CRC but also served an essential role in the CRC initiation and progression.

## Discussion

Colorectal adenocarcinoma is one of the most common cancers globally, and its incidence rate is high. Therefore, the research on the tumor metastasis mechanism of colorectal adenocarcinoma is of great significance to explore new targets and improve the therapeutic effect and prognosis of patients with colorectal adenocarcinoma. Compared to using a single analysis or database, this study performed a more detailed and more effective analysis, that is, screening hub genes based on WGCNA and differential gene expression analysis in TCGA and GEO database. Differential gene expression analysis 82 critical genes with the same expression trends were identified in TCGA and GSE25070 databases by bioinformatics analysis. Functional annotation of these genes demonstrated that they were involved in bicarbonate transport and organic anion transport, which can alter the intestinal microenvironment to affect the initiation and progression of tumors.

Furthermore, based on the cytoHubba plug-in in Cytoscape, 10 genes (SLC26A3, GUCA2A, CLCA4, MS4A12, GUCA2B, CLCA1, AQP8, SI, KRT20, and SLC4A4) were screened. The down expression of five genes (SLC26A3, GUCA2A, CLCA4, CLCA1, and AQP8) was significantly associated with poor overall survival in colorectal cancer adenocarcinoma patients, and patients with lower expression levels of CLCA1 had poorer disease-free survival rates. Subsequently, clinical correlation analysis and the immunohistochemical analysis of the five hub genes were analyzed, revealing these genes’ potential as novel prognostic biomarkers.

In addition, none of the single biomarkers can be used to detect cancer and achieve the required specificity and sensitivity in the cancer research along (Muinao et al., 2019), and multiple biomarkers have more advantages than a single biomarker. Therefore, we constructed a 3-gene risk model to predict the prognosis of colorectal adenocarcinoma, and subsequent studies suggested the 3-gene risk model has a good prognostic value. Furthermore, the prognostic model showed an excellent predictive efficiency compared with other published models.

Like all studies, our study has limitations in classifying different subtypes of tumors. Our study is conducted without the colorectal adenocarcinoma subtype. In addition, the specific pathogenesis and molecular targets need to be further verified by a series of experiments.

In conclusion, by integrating WGCNA and differential gene expression analysis, our study screened five important survival-related genes (SLC26A3, GUCA2A, CLCA4, CLCA1, and AQP8) and a 3-gene risk model with the potential to predict the prognosis of colorectal adenocarcinoma.

## Data Availability

Publicly available datasets were analyzed in this study. These data can be found at: the TCGA database (https://portal.gdc.cancer.gov/) and GEO database (https://www.ncbi.nlm.nih.gov/geo/).
